# Supplementary material for: Content-rich biological network constructed by mining PubMed abstracts
Source: BMC Bioinformatics. 2004 Oct 8;5:147. doi: 10.1186/1471-2105-5-147 (PMC528731; doi:10.1186/1471-2105-5-147)
Supplement: Additional File 2 — The original results of the above study (non-essential files are deleted to keep the file size under the limit set by BMC bioinformatics). [file 1471-2105-5-147-S2.bz2 › chilibotAdditionalFile2/dip10/658ID10525412E11049/html/NIFD_NIFK.html]

 


 **NIFD** and **NIFK** 
  
Found 195 abstracts in PubMed, retrieved 10.  
 

 What does Google say? 
 PDF only 
| .edu only 

---

**Interactive relationship** (e.g. stimulation, inhibition, etc)

**Non-interactive relationship** (e.g. studied together, co-existance, homology, etc.)

- VnfY is required for full activity of the vanadium containing dinitrogenase  [ **NIFK** ]   [ **NIFD** ]  in Azotobacter vinelandii.  Ref: 12644512 J Bacteriol, 2003
- These results indicate that VnfY has a role in the maturation of the V dependent dinitrogenase  [ **NIFK** ]   [ **NIFD** ] , with a specific role in the formation of the V containing cofactor and or its insertion into apodinitrogenase.  Ref: 12644512 J Bacteriol, 2003
- Our dinitrogenase  [ **NIFK** ]   [ **NIFD** ]  gene nifH sequences grouped together with a group of cyanobacteria from the subtropical North Pacific.  Ref: 12406777 Appl Environ Microbiol, 2002
- The MoFe protein component of the complex metalloenzyme nitrogenase is an alpha2beta2 tetramer encoded by the  **nifD**  and the  **nifK**  genes.  Ref: 12468552 J Biol Chem, 2003
- Under salt stress, the amount of dinitrogenase  [ **NIFK** ]   [ **NIFD** ]  reductase inactivated by ADP ribosylation was strongly reduced, indicating that the dinitrogenase  [ **NIFK** ]   [ **NIFD** ]  reductase ADP ribosyl transferase DRAT activity was also inhibited by increased NaCl concentrations.  Ref: 11927988 Curr Microbiol, 2002
- As such this represents a new fold capable of binding FeMo co, where the only previous example was that seen in dinitrogenase  [ **NIFK** ]   [ **NIFD** ] .  Ref: 12754195 J Biol Chem< MedlineTA>J Biol Chem, 2002
- The dinitrogenase  [ **NIFK** ]   [ **NIFD** ]  component proteins of the conventional Mo nitrogenase MoFe protein and of the alternative Fe only nitrogenase FeFe protein were both isolated and purified from Rhodobacter capsulatus, redox titrated according to the same procedures and subjected to an EPR spectroscopic comparison.  Ref: 11895435 Eur J Biochem, 2002
- A mutant strain having an insertion mutation in vnfY has 10 fold less vnf dinitrogenase  [ **NIFK** ]   [ **NIFD** ]  activity and exhibits a greatly diminished level of 49 V label incorporation into the V dependent dinitrogenase  [ **NIFK** ]   [ **NIFD** ]  when compared to the wild type.  Ref: 12644512 J Bacteriol, 2003
- As another level of control, nitrogenase activity was regulated by at least three types of mechanisms in strain BH72 covalent modification of dinitrogenase  [ **NIFK** ]   [ **NIFD** ]  reductase NifH, probably by ADP ribosylation, and two other, unknown means.  Ref: 11914357 J Bacteriol, 2002
- Although restriction analysis of the  **nifD**   **nifK**  intergenic DNA fragment confirmed the unique nature of Rhizobium sp.  Ref: 12802480 Arch Microbiol< MedlineTA>Arch Microbiol, 2002
- These symbionts are thought to provide enzymes, e.g., cellulase and dinitrogenase  [ **NIFK** ]   [ **NIFD** ] , which assist the host in utilizing wood as a primary food source.  Ref: 12450854 Appl Environ Microbiol, 2002
- Another novel S = 1 2 signal at g = 1.9.92 and 1.77 was observed on further, enzymatic reduction of the dithionite reduced state of Rc1Fe with the dinitrogenase  [ **NIFK** ]   [ **NIFD** ]  reductase component Rc2Fe of the same enzyme system turnover conditions in the presence of N2 and ATP.  Ref: 11895435 Eur J Biochem, 2002
- The Fe only nitrogenase and the Mo nitrogenase from Rhodobacter capsulatus a comparative study on the redox properties of the metal clusters present in the dinitrogenase  [ **NIFK** ]   [ **NIFD** ]  components.  Ref: 11895435 Eur J Biochem, 2002
- Significant amounts of both dinitrogenase  [ **NIFK** ]   [ **NIFD** ]  and dinitrogenase  [ **NIFK** ]   [ **NIFD** ]  reductase were only formed when the growth medium was supplemented with molybdate 1 microM.  Ref: 12667075 Biochemistry, 2003
